# Suture Fixation Versus Screw Fixation in Pediatric Tibial Eminence Fractures: A Systematic Review and Meta-Analysis of Clinical Outcomes and Reoperation Rates

**DOI:** 10.7759/cureus.99853

**Published:** 2025-12-22

**Authors:** Ahmed Elnewishy, Ziad El Menawy, Mohamed Zahed, Mahmoud Elmesalmi, Nour Elnaggar, Farouk Ahmed, Mahmoud Odeh, Mohamed Elgamal

**Affiliations:** 1 Trauma and Orthopedics, Royal Berkshire Hospital, Reading, GBR; 2 Trauma and Orthopedics, University Hospital of Wales, Cardiff, GBR; 3 Pediatric Burns and Plastic Surgery, Royal Manchester Children’s Hospital, Manchester, GBR; 4 Trauma and Orthopedics, Manchester Royal Infirmary, Manchester, GBR; 5 Internal Medicine, Zayed Military Hospital, Abu Dhabi, ARE; 6 Orthopedics, John Radcliffe Hospital, Oxford University Hospitals NHS Trust, Oxford, GBR; 7 Trauma and Orthopedics, St George's University Hospitals NHS Foundation Trust, London, GBR; 8 Medicine, Zagazig University, Zagazig, EGY; 9 Emergency Medicine, Queen Alexandra Hospital, Portsmouth, GBR; 10 Trauma and Orthopedics, Cardiff and Vale University Health Board, Cardiff, GBR; 11 Trauma and Orthopedics, Southend Hospital, Southend-on-Sea, GBR

**Keywords:** anterior cruciate ligament (acl), arthroscopic fixation, hardware removal, pediatric knee injury, pediatric tibial eminence, return to sport, suture fixation, tibial eminence fracture, tibial spine avulsion

## Abstract

Suture fixation (SF) has gained prominence as a physeal-sparing alternative to screw fixation (SCF) in the operative management of pediatric tibial eminence fractures, aiming to reduce hardware-related complications while maintaining joint stability. This meta-analysis evaluated clinical and functional outcomes comparing SF with SCF in skeletally immature patients. A systematic review of comparative studies was conducted following the Preferred Reporting Items for Systematic reviews and Meta-Analyses (PRISMA) guidelines. Outcomes assessed included clinically relevant postoperative measures used to evaluate overall treatment success. Fixed-effect models were used to generate pooled effect estimates. Heterogeneity was quantified using the I² statistic, and publication bias was examined with funnel plots and Egger’s test. Four studies encompassing 224 pediatric patients were included. SF resulted in a significantly lower reoperation frequency (OR = 0.22, 95% CI: 0.12-0.42, p < 0.00001, I² = 42%) and markedly reduced hardware removal (OR = 0.08, p < 0.00001, I² = 0%). Return to sport rates were superior in the SF group (OR = 2.71, p = 0.02, I² = 0%). No significant differences were observed between SF and SCF for postoperative instability (OR = 0.72, p = 0.50, I² = 0%), arthrofibrosis requiring surgery (OR = 0.80, p = 0.60, I² = 0%), or full range of motion recovery (OR = 1.36, p = 0.34, I² = 0%). Publication bias was not detected. Compared with SCF, SF provides substantially lower rates of reoperation and hardware removal while maintaining equivalent stability, stiffness outcomes, and functional recovery. SF should be strongly considered the preferred technique in appropriately selected pediatric patients.

## Introduction and background

Tibial eminence fractures (TEFs), also known as tibial spine fractures or anterior cruciate ligament (ACL) avulsion fractures, account for nearly 14% of ACL-related injuries in children [1]. Although they constitute only 2-5% of traumatic knee effusions, TEFs are considered a significant clinical entity. The affected population predominantly comprises skeletally immature males aged 8-14 years, representing 66-70% of cases [2]. In the largest multicenter epidemiological study of 661 pediatric cases across eight institutions, the median age at injury was 12.2 years, confirming this concentration in early adolescence [2].

Biomechanically, the mechanism of TEFs is similar to ACL rupture in adults. In immature knees, incomplete ossification of the tibial spine renders the bone more susceptible to avulsion than the ligament itself [3]. TEFs account for 70.7% of proximal tibial injuries in children, as demonstrated by a recent systematic review of pediatric proximal tibial fractures [4].

Fractures of the tibial eminence occur during a period of unique structural and physiological characteristics of the growing knee [5]. The ACL inserts on the tibial eminence, located on the proximal tibial plateau, which is not fully ossified in children. This biomechanical weakness makes the bone fragment more likely to avulse under tensile or rotational stress than the ligament. The open physes (growth plates) also influence both the injury mechanism and surgical management. Implant penetration carries the risk of physeal injury and potential growth disturbance due to the close proximity of the tibial eminence to the proximal tibial physis [6]. Consequently, physeal-sparing fixation techniques, particularly all-suture or hybrid constructs, are more commonly employed [7].

Immature knees exhibit increased ligamentous elasticity and decreased subchondral bone stiffness, predisposing them to this type of avulsion pattern [8]. Fixation studies comparing suture and screw techniques suggest that skeletal maturity influences fixation strength: in immature bone, suture and screw techniques are comparable, whereas mature specimens may demonstrate greater resistance with sutures [9]. Arthroscopic, physeal-sparing techniques, such as suture pull-through or tri-pulley configurations, have demonstrated favorable outcomes [10].

ACL insertion avulsion is classically defined as a tibial spine fracture. The Meyers and McKeever classification system standardizes tibial spine avulsions based on the extent of fragment separation, guiding treatment decisions [11]. Type I fractures are nondisplaced with an intact posterior fragment. Type II fractures demonstrate beak-like anterior elevation [12]. Complete avulsion is classified as Type III, with further subclassification: Type IIIA indicates maintained anatomic position, whereas Type IIIB denotes rotation or inversion [13]. Type IV, added by Zaricznyj in 1977, includes comminuted or rotated fragments, usually following high-energy trauma [14]. MRI refinements have increased diagnostic accuracy by identifying meniscal entrapment or chondral injury not visible on plain radiographs [15]. Epidemiological studies indicate that Types II and III comprise over 80% of pediatric tibial spine injuries, most of which require operative management.

Insufficient reduction or fixation may leave the ACL elongated, causing persistent anterior laxity. Even after union, some patients experience functional instability and reduced athletic performance [16]. The most common postoperative complication is arthrofibrosis, a fibrotic contracture limiting motion, occurring in up to 30% of cases with prolonged immobilization. Early rehabilitation and range of motion (ROM) exercises greatly reduce this risk [17]. Other complications include mechanical impingement or an extension block from a prominent fragment, nonunion or malunion with angular deformity or limb-length discrepancy, and early osteoarthritic degeneration, with 18% of inadequately managed cases showing radiographic changes [18-20].

Arthroscopic suture fixation (SF) uses nonabsorbable sutures (e.g., FiberWire, ETHIBOND) passed through the ACL and secured via transtibial tunnels or anchors. This approach provides anatomic reduction and tensioning without intra-articular hardware, avoids physeal injury, and eliminates the need for implant removal. Biomechanical analyses demonstrate comparable or superior ultimate failure loads compared with screw fixation (SCF), permitting early mobilization and less postoperative stiffness [21]. Both techniques yield similar outcomes in immature knees, but sutures have fewer hardware-related complications [22].

SCF involves rigid cannulated or headless compression screws, which allow early rehabilitation. Advances such as smaller screw diameters (3-5 mm) and bioabsorbable materials have reduced hardware impingement and, in select cases, obviated the need for removal [23,24]. Comparative meta-analyses show that screws and SF provide similar stability and function, but screws carry a higher reoperation rate due to hardware-related complications [25,26]. More recent evidence indicates that SF is associated with superior Lysholm and IKDC scores and fewer postoperative issues, whereas screws are associated with shorter operative times but increased removal rates [27,28].

This systematic review and meta-analysis integrates current evidence comparing suture constructs and SCF, aiming to assess clinical and functional outcomes in children with TEFs.

## Review

Methods

Search Strategy

A comprehensive literature search was conducted in November 2025 across PubMed, Scopus, Web of Science, Embase, and the Cochrane Library to identify studies comparing SF and SCF for the management of pediatric TEFs. The search strategy combined Medical Subject Headings (MeSH) and free-text terms, including “tibial eminence fracture”, “tibial spine avulsion”, “intercondylar eminence fracture”, “suture fixation”, “transosseous suture”, “screw fixation”, “arthroscopic fixation”, and “pediatric knee trauma”. Reference lists of all eligible full-text studies and relevant systematic reviews were manually screened to identify any additional suitable publications.

Inclusion Criteria

Studies were included if they involved pediatric patients with open physes and Type II or III TEFs according to the Meyers-McKeever classification. Only comparative observational studies, either prospective or retrospective, directly comparing SF with SCF were considered. Eligible studies needed to report sufficient outcome data to allow meta-analysis, including postoperative functional outcomes, reoperation rates for instability, arthrofibrosis, hardware removal, or other re-interventions. Only articles published in English and available in full text were included.

Exclusion Criteria

Studies were excluded if they lacked a direct comparison between suture and SCF or did not provide extractable numerical outcomes for relevant endpoints. Additional exclusion criteria included studies with skeletally mature participants or mixed-age cohorts without pediatric subgroup stratification, single-arm case series, case reports, cadaveric or biomechanical studies, surgical technique descriptions, reviews, commentaries, or conference abstracts. In instances of overlapping patient datasets, the study with the most complete and clearly reported cohort was selected.

A summary of the inclusion and exclusion criteria is provided in Table 1.

**Table 1:** Inclusion and exclusion criteria for study selection ROM, range of motion; RTS, return to sport; SCF, screw fixation; SF, suture fixation; TEF, tibial eminence fracture

Category	Criteria
Inclusion criteria	Meyers-McKeever Type II or III TEFs in children with open physes
	Comparative studies evaluating SFS and SCF
	Retrospective or prospective observational comparative studies
	Studies reporting extractable postoperative outcomes (reoperation, instability, arthrofibrosis, hardware removal, ROM, and RTS)
	Full-text articles published in English
Exclusion criteria	Adult or skeletally mature participants
	Mixed-age cohorts without separate pediatric analysis
	Single-arm case series or case reports
	Cadaveric or biomechanical studies
	Technical notes, reviews, or commentaries
	Conference abstracts without full data
	Studies lacking extractable numerical outcomes
	Overlapping datasets (less complete dataset excluded)

Outcome Measures

The primary outcomes of interest were reoperation rate, hardware removal rate, postoperative knee instability, and arthrofibrosis requiring surgical intervention, as these represent the most clinically significant complications following operative fixation. Secondary outcomes included return to sport (RTS) and full ROM recovery, which reflect functional recovery. Collected demographic variables, details of the fixation technique, and follow-up duration provided context for interpreting treatment effects across studies.

Data Extraction and Quality Assessment

Data were extracted using predesigned standardized forms to record study characteristics, patient demographics, fracture classification, fixation technique, follow-up duration, and all reported postoperative outcomes. Extraction was performed independently and cross-checked for accuracy, with disagreements resolved by consensus. The methodological quality of included studies was assessed using the Newcastle-Ottawa Scale (NOS), which evaluates study selection, comparability of intervention groups, and reliability of outcome assessment [29]. Each study received a total NOS score and was classified as low, moderate, or high quality according to established criteria.

Statistical Analysis

All statistical analyses were performed using Review Manager (RevMan v5.4, The Cochrane Collaboration) [26]. ORs with 95% CIs were used to pool results for dichotomous outcomes. Fixed-effect or random-effects models were chosen based on heterogeneity. When heterogeneity was low (I² < 50% on the chi-square test), a fixed-effect model was adopted. Egger’s regression method and visual inspection of funnel plots were used to assess publication bias, with a two-tailed p-value < 0.05 considered statistically significant.

Results

Search Result and Study Selection

A comprehensive systematic search was conducted to identify studies comparing SF and SCF for the management of pediatric TEFs. The initial search across all databases and reference lists yielded 176 records. After removing 41 duplicates, 135 unique studies remained for title and abstract screening. During this phase, 113 records were excluded for not meeting the predefined eligibility criteria. The most common reasons for exclusion were non-comparative study designs, lack of a direct SF-SCF comparison, adult or mixed-age populations without pediatric stratification, single-arm case series, biomechanical or cadaveric studies, technical notes, reviews, and studies without extractable clinical outcomes.

Following this stage, 22 full-text articles were assessed for eligibility. After detailed evaluation, 18 studies were excluded due to reasons such as absence of a direct SF-SCF comparison, insufficient or non-extractable data, inadequate methodology or follow-up, or inclusion of a skeletally mature population. Ultimately, four studies met all inclusion criteria and were incorporated into the final qualitative and quantitative synthesis.

**Figure 1:**
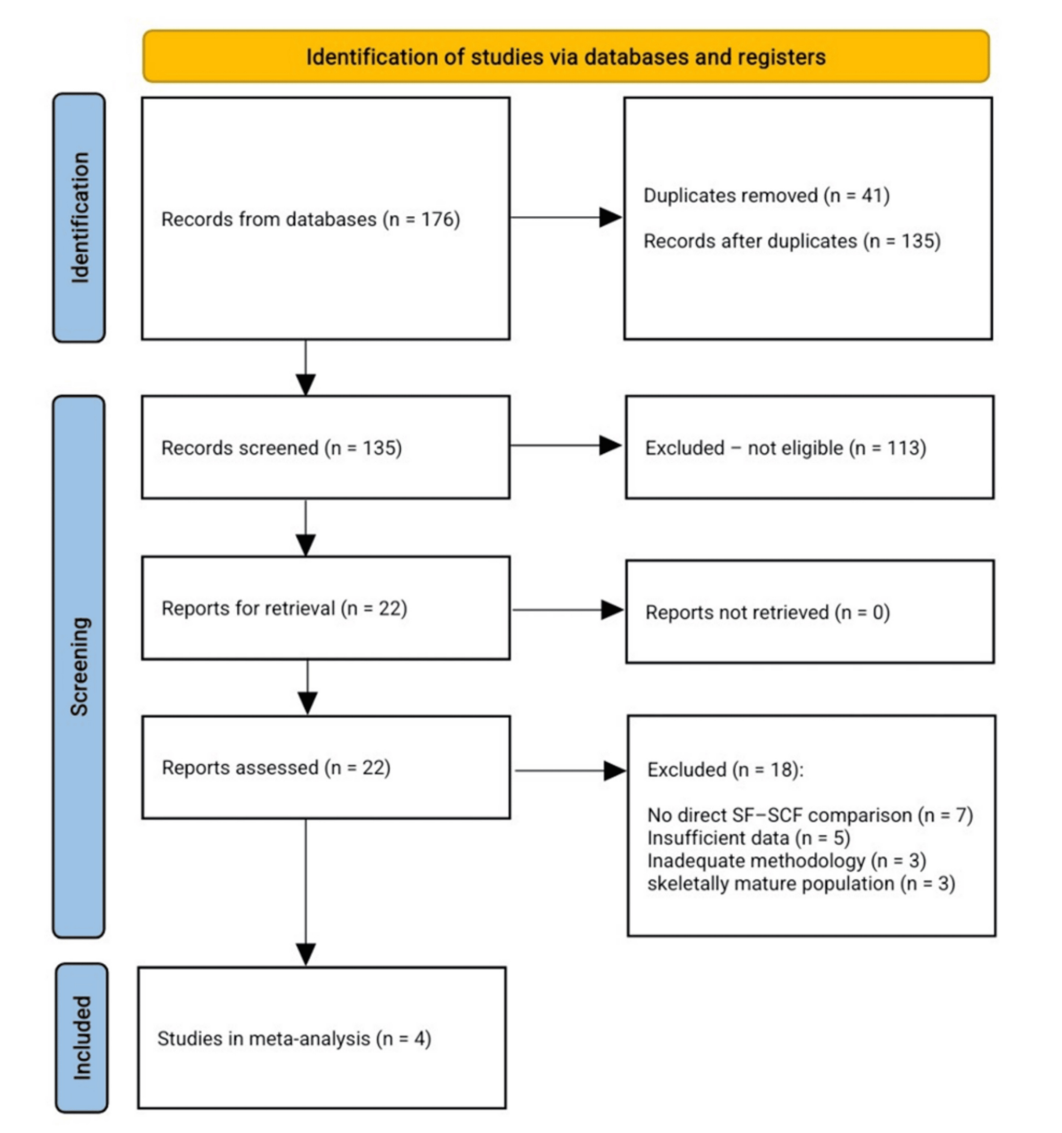
PRISMA flowchart for the included studies PRISMA, Preferred Reporting Items for Systematic reviews and Meta-Analyses; SCF, screw fixation; SF, suture fixation

The complete study selection process is presented in Figure 1.

Study Characteristics

The four studies included in the final synthesis collectively evaluated 224 pediatric patients with TEFs managed operatively using either SF or SCF. Among these patients, 112 underwent suture-based fixation, and 112 received screw-based fixation. All studies focused exclusively on skeletally immature patients with Meyers-McKeever type II or III tibial spine avulsion injuries and provided comparable demographic and clinical details. The mean age across cohorts ranged from 10.9 to 11.8 years.

Despite variation in sample size and follow-up duration, all studies consistently reported extractable outcomes, including radiographic union, postoperative knee stability, ROM, RTS, complications such as arthrofibrosis or hardware-related symptoms, and the need for reoperation. These characteristics enabled a structured comparison of clinical and functional results between suture- and screw-based fixation techniques.

A detailed summary of the key characteristics of the included studies is presented in Table 2.

**Table 2:** Summary of key methodological and clinical characteristics of studies comparing SF and SCF for pediatric TEFs ACL, anterior cruciate ligament; FU, follow-up; HF, hybrid fixation; HSF, headless screw fixation; IKDC, International Knee Documentation Committee score; LOA, lysis of adhesions; MUA, manipulation under anesthesia; QoL, quality of life; ROM, range of motion; RTP, return to preinjury level of sport; RTS, return to sport; SF, suture fixation; SCF, screw fixation; TSA, tibial spine avulsion

Category	Callanan et al. [30]	Ercan et al. [31]	Granadillo [32]	Jääskelä et al. [33]
Study design	Retrospective cohort, single pediatric institution (2000-2014)	Retrospective cohort (2015-2020)	Retrospective comparative case series (2000-2012)	Multicenter pediatric/young adolescent cohort
Sample size	33 sutures/35 screws	11 sutures/13 headless screws	36 sutures/35 screws (+7 hybrid)	32 open sutures/29 screws
Level of evidence	III	III	III	III
Patient demographics	Mean age 11.8; 72% male; type II 22%, type III 78%; meniscal entrapment 31%; meniscal tear 22%	Median age 11; similar sex distribution; minimal associated injuries; 3 meniscal tears repaired	Mean age 11.4; 68% male; 78% sports mechanism; meniscal injury predicted stiffness	Mean age 11.2; 57% male; type II-III; 14.8% concomitant injuries
Intervention details	Suture = tibial tunnels; screw = epiphyseal cannulated screw	Suture = Ultrabraid + tibial tunnels; screw = headless compression screw	Suture = transosseous tunnels; screw = cannulated screws/SmartNails	Suture = open osteosuture; screw = arthroscopic epiphyseal screws
Follow-up	Median 26 months	Minimum 2 years; median 34-42 months	Minimum 6 months; many long-term	Mean 87 months (24-189)
Outcome measures	ROM, instability, RTS, radiographic union, fragment elevation, reoperations	ROM, KT-1000 stability, IKDC, Lysholm, Tegner, strength, union time	ROM loss, residual pain, instability, union time, RTS, reoperations	RTP level, IKDC, QoL, displacement, regression predictors, reoperations
Results	Union: 3.2 mo vs 5.3 mo (P = 0.03). Fragment elevation: 5.4 vs 3.5 mm (P = 0.005). Full ROM: 76% vs 66%. Arthrofibrosis requiring surgery: 24% vs 31%. Instability: 0% vs 6%. RTS: 91% vs 74% (NS). Reoperations: 39% vs 66% (P = 0.03). Implant removal: 9% vs 62% (P < 0.001)	Operating time: 95 vs 65 min (P = 0.007). Union: 74 vs 72 days (NS). ROM: minimal deficits; no arthrofibrosis. Lysholm, Tegner, and IKDC scores similar. Stability: Lachman/pivot 0-1; KT-1000 <3 mm. Strength: symmetric. Daily activity resumed after 6 months	Whole cohort: flexion loss 22%, extension loss 42%; RTS 96%; healing ~19 weeks; mild pain 23%; instability 14%; complications 33%. Suture vs screw: tourniquet time ↑ in suture (P = 0.002). Healing 19 vs 19.7 weeks (NS). RTS 100% vs 93% (NS). Residual symptoms similar. Instability 14% both. Meniscal injury → stiffness (P = 0.002)	RTP: preinjury level 81.2% (suture) vs 69.0% (screw) (NS). Any level: 90.6% vs 89.7%. Time to RTP: 8 weeks vs 21 weeks (P < 0.05). KT-1000 >3 mm (OR 15.2) → failure to RTP; reoperation → poor IKDC (OR 19.0)
Complications	Arthrofibrosis requiring LOA/MUA: 24% vs 34%. ACLR 9% both. Meniscal repeat surgery 6-9%. High screw burden: removal 62%, multiple reoperations	No complications; no reoperations; no hardware removals	At least one complication: 39% vs 49% (NS). Arthrofibrosis requiring surgery: 8% vs 14%. Hardware removal: 11% vs 29%. Reinjury: 8% vs 14%	Reoperations: 9.4% vs 20.7%. Screw: pain → removal; instability → ACLR; displacement → repeat fixation. Minimal arthrofibrosis overall
Conclusion	Equivalent clinical outcomes, but screw = far higher reoperation and hardware removal → suture preferred	Suture and headless screw equally effective; headless screw shortens surgery and avoids a second procedure	No major differences; meniscal injury strongest predictor of stiffness; screw more likely to need removal	Both effective long-term; open suture = faster RTP and fewer failures; screw increases risk of poor RTP if reduction imperfect

Quality Assessment of the Included Studies

Study quality was evaluated using the NOS, which assesses methodological rigor across three key domains: cohort selection, comparability of groups, and outcome assessment. Each study was assigned a rating based on the number of stars awarded in each domain. Studies were subsequently categorized as low, moderate, or high quality according to their total NOS scores. Table 3 presents the detailed quality assessment for each included study.

**Table 3:** Quality assessment of included studies using the NOS ★ indicates a low score for the respective category. ★★ represents a moderate score, reflecting acceptable methodological quality. ★★★ denotes a high score, indicating strong quality in the respective domain. NOS, Newcastle-Ottawa Scale

Study	Selection	Comparability	Outcome	Total score (out of 9)
Callanan et al. [30]	★★★★	★★	★★	8
Ercan et al. [31]	★★★	★★	★★★	8
Granadillo [32]	★★★★	★★	★★	8
Jääskelä et al. [33]	★★★★	★★	★★★	9

Results of the meta-analysis

Comparison of Reoperation Rates Between SF and SCF in Pediatric TEFs

A forest plot analysis of reoperation rates in pediatric TEFs treated with SF and SCF demonstrated a statistically significant lower reoperation rate for SF (pooled OR 0.22, 95% CI: 0.12-0.42, p < 0.00001), corresponding to a 79% lower risk of reoperation for children treated with SF compared with SCF.

**Figure 2:**
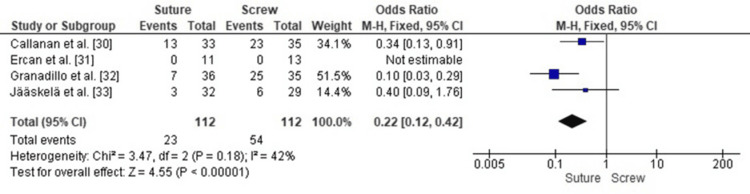
Forest plot comparing SF and SCF for reoperation rates SCF, screw fixation; SF, suture fixation Sources: [30-33]

Heterogeneity across the included studies was low (I² = 42%, p = 0.18), indicating minimal variability in study designs, populations, or fixation techniques. This enhances the validity of the pooled effect, demonstrating that the reduced risk of reoperation with SF is a robust and reproducible finding across the available evidence (Figure 2).

Publication Bias Assessment for Reoperation Rate

**Figure 3:**
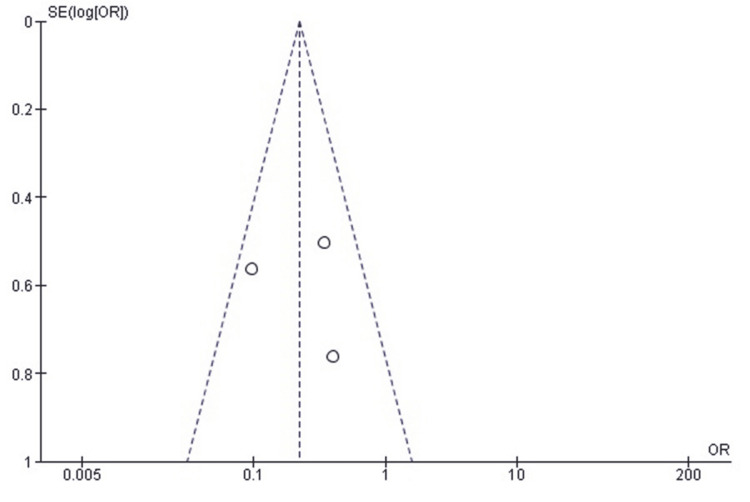
Funnel plot assessing publication bias for studies comparing SF and SCF regarding reoperation rates SCF, screw fixation; SF, suture fixation

A funnel plot of the effect estimates (Figure 3) showed that the distribution of effect estimates was roughly symmetrical. Minor asymmetry was likely attributable to differences in study size and standard error rather than selective reporting or systematic publication bias. Egger’s regression test was not statistically significant (p > 0.05), suggesting that the observed pattern is consistent with random variation and not due to small-study effects.

Comparison of Hardware Removal Rates Between SF and SCF in Pediatric TEFs

Forest plot analysis comparing hardware removal rates between suture and SCF demonstrated a statistically significant difference, with SCF associated with a markedly higher need for hardware removal. The pooled OR was 0.08 (95% CI: 0.03-0.19, p < 0.00001), indicating that children treated with SF were 92% less likely to require hardware removal compared with those treated with SCF.

**Figure 4:**
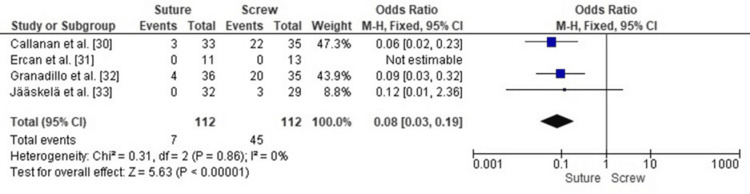
Forest plot comparing SF and SCF for hardware removal rates SCF, screw fixation; SF, suture fixation Sources: [30-33]

Heterogeneity across the included studies was very low (I² = 0%, p = 0.86), indicating highly consistent effect estimates across study designs and populations. This confirms that the reduced hardware removal burden with SF is a robust finding across the available evidence (Figure 4).

Publication Bias Assessment for Hardware Removal Rate

**Figure 5:**
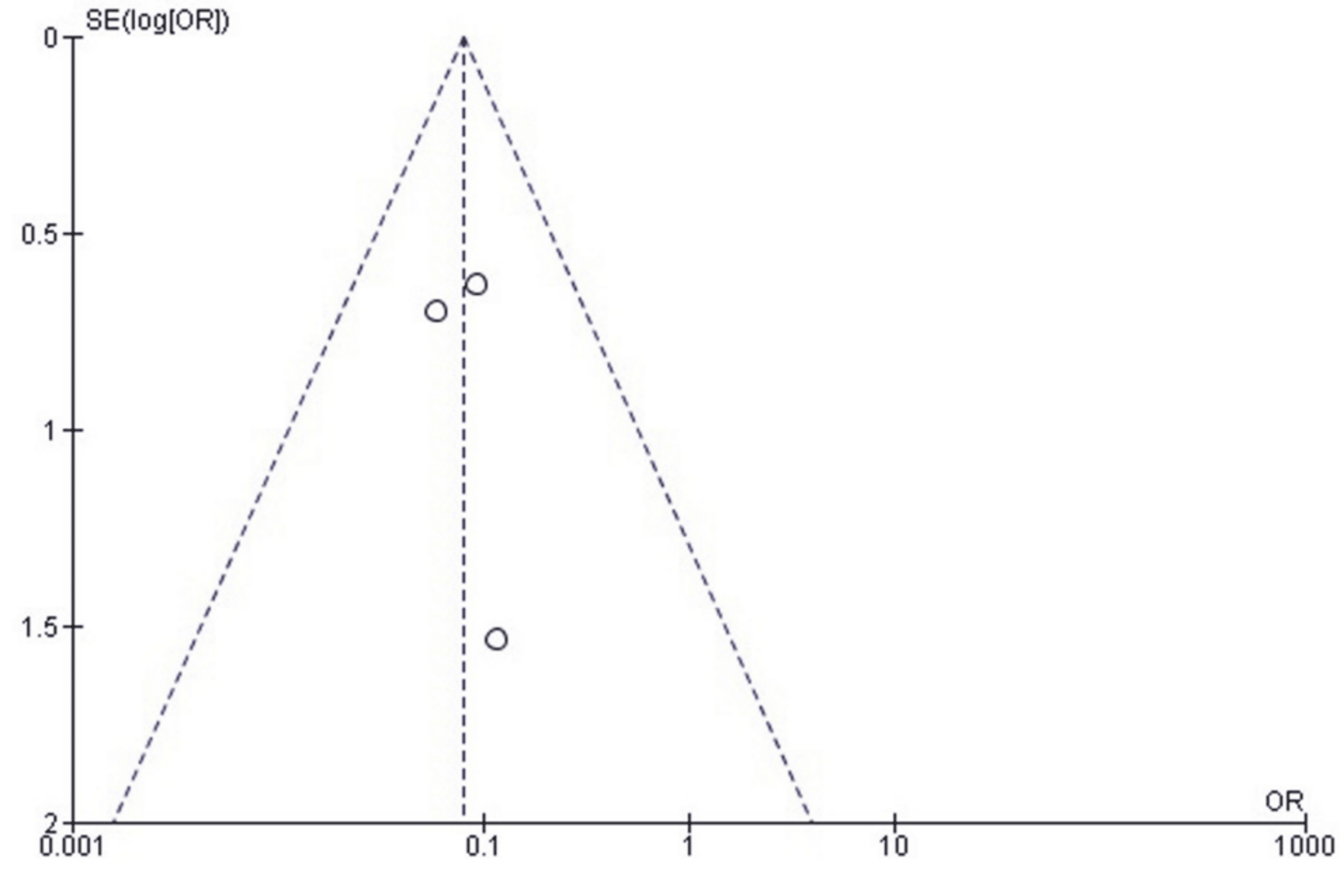
Funnel plot assessing publication bias for studies comparing SF and SCF regarding hardware removal rates SCF, screw fixation; SF, suture fixation

A funnel plot was used to evaluate publication bias, showing approximately symmetrical study estimates. Minor asymmetry was likely due to variation in standard error and sample size rather than publication bias (Figure 5). Egger’s regression test was not statistically significant (p > 0.05), supporting the interpretation that any asymmetry is random rather than due to bias or small-study effects.

Comparison of Postoperative Knee Instability Between SF and SCF in Pediatric TEFs

**Figure 6:**
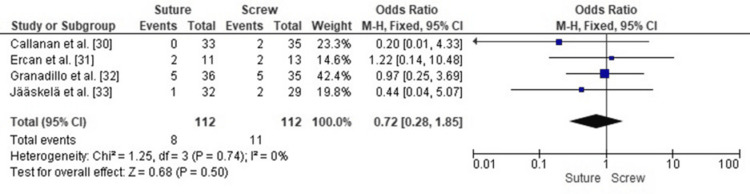
Forest plot comparing SF and SCF for postoperative knee instability SCF, screw fixation; SF, suture fixation Sources: [30-33]

A forest plot analysis comparing postoperative knee instability in pediatric TEFs treated with SF versus SCF showed no statistically significant difference between the two fixation techniques (pooled OR 0.72, 95% CI: 0.28-1.85, p = 0.50). Heterogeneity across the included studies was negligible (I² = 0%, p = 0.74), indicating consistent effect estimates across study populations and methodologies, enhancing the reliability of the pooled analysis, and confirming that the similarity in instability rates between SF and SCF was not due to variability in the evidence (Figure 6).

Publication Bias Assessment for Postoperative Knee Instability

**Figure 7:**
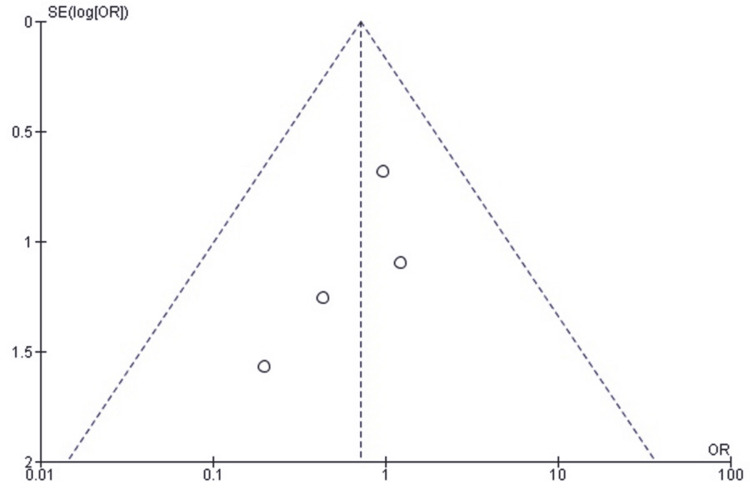
Funnel plot assessing publication bias for studies comparing SF and SCF regarding postoperative knee instability SCF, screw fixation; SF, suture fixation

A funnel plot indicated an approximately symmetrical distribution of study estimates (Figure 7), suggesting any minor asymmetry was likely due to differences in standard error and small sample sizes rather than systematic publication bias. Egger’s regression test showed no statistically significant evidence of publication bias (p > 0.05).

Comparison of Arthrofibrosis Requiring Surgery Between SF and SCF in Pediatric TEFs

**Figure 8:**
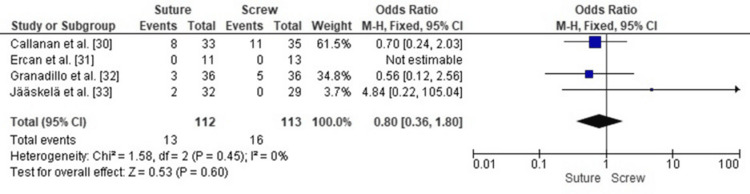
Forest plot comparing SF and SCF for arthrofibrosis requiring surgery SCF, screw fixation; SF, suture fixation Source: [30-33]

Forest plot analysis comparing rates of arthrofibrosis requiring surgical intervention revealed no statistically significant difference between SF and SCF (pooled OR 0.80, 95% CI: 0.36-1.80, p = 0.60). Heterogeneity was extremely low (I² = 0%, p = 0.45), indicating stable effect estimates across populations and surgical techniques, supporting the conclusion that neither method offers a significant advantage in preventing postoperative arthrofibrosis requiring operative management (Figure 8).

Publication Bias Assessment for Arthrofibrosis Requiring Surgery

**Figure 9:**
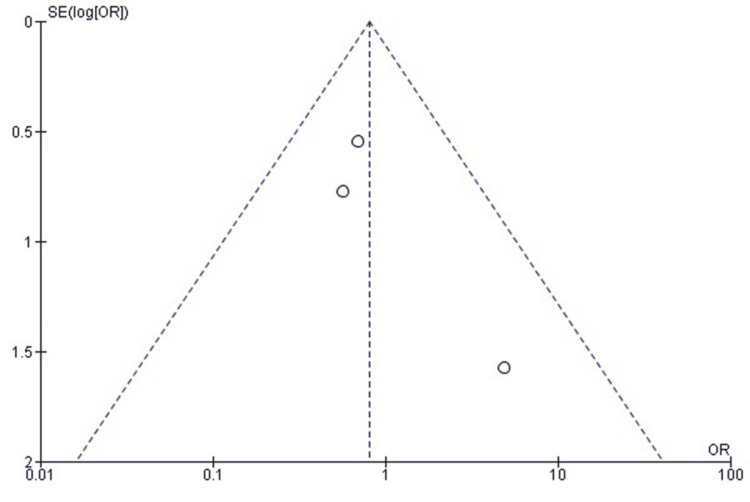
Funnel plot assessing publication bias for studies comparing SF and SCF regarding arthrofibrosis requiring surgery SCF, screw fixation; SF, suture fixation

A funnel plot was used to assess publication bias and demonstrated an approximately symmetrical distribution of the study effect estimates (Figure 9). This symmetry is most likely attributable to differences in sample size and standard error among the included studies rather than selective outcome reporting. Egger’s regression test showed no statistically significant evidence of publication bias (p > 0.05), further supporting that the observed distribution reflects random variation rather than small-study effects or systematic bias.

Comparison of RTS Between SF and SCF in Pediatric TEFs

**Figure 10:**
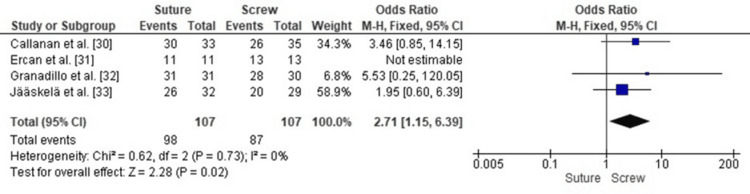
Forest plot comparing SF and SCF for RTS RTS, return to sport; SCF, screw fixation; SF, suture fixation Sources: [30-33]

Forest plot analysis comparing RTS in patients treated with SF versus SCF for pediatric TEFs demonstrated a statistically significant advantage for SF (OR 2.71; 95% CI: 1.15-6.39, p = 0.02). Heterogeneity across the included studies was very low (I² = 0%, p = 0.73), indicating a consistent effect estimate across different clinical settings, populations, and study designs. This consistency reinforces the reliability of the pooled findings and supports the conclusion that SF more consistently facilitates return to athletic activity following tibial spine avulsion repair (Figure 10).

Publication Bias Assessment for RTS

**Figure 11:**
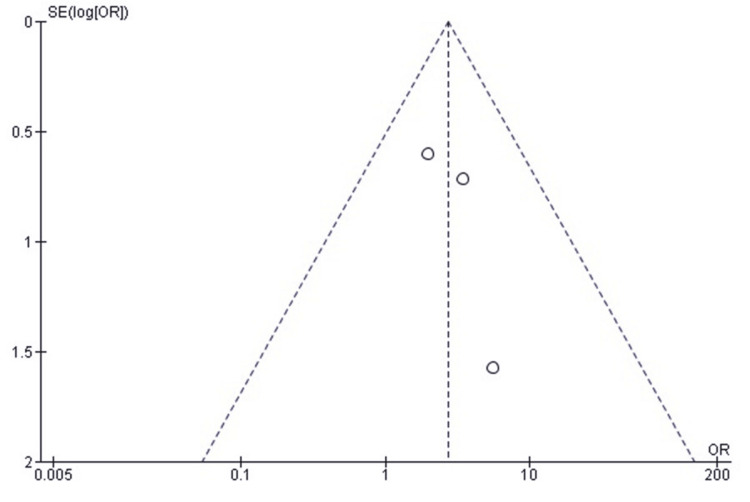
Funnel plot assessing publication bias for studies comparing SF and SCF regarding RTS RTS, return to sport; SCF, screw fixation; SF, suture fixation

Publication bias was evaluated using a funnel plot, which showed an approximately symmetrical distribution of study estimates (Figure 11). Minor asymmetry was observed, likely due to variations in sample size and standard errors rather than true publication bias. Egger’s regression test showed no statistically significant evidence of publication bias (p > 0.05).

Comparison of Full ROM Recovery Between SF and SCF in Pediatric TEFs

A forest plot analysis comparing ROM recovery for SF versus SCF in pediatric TEFs showed no statistically significant difference between the two fixation methods (pooled OR 1.36, 95% CI: 0.72-2.57, p = 0.34).

**Figure 12:**
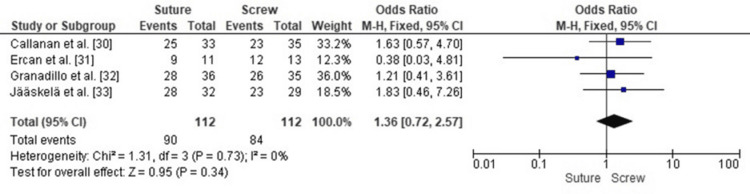
Forest plot comparing SF and SCF for full ROM recovery ROM, range of motion; SCF, screw fixation; SF, suture fixation Sources: [30-33]

Heterogeneity across the included studies was low (I² = 0%, p = 0.73), indicating that the results were consistent across study designs, populations, and surgical approaches (Figure 12). Therefore, neither fixation method demonstrated a clear advantage in restoring full knee ROM following surgical repair.

Publication Bias Assessment for Full ROM Recovery

**Figure 13:**
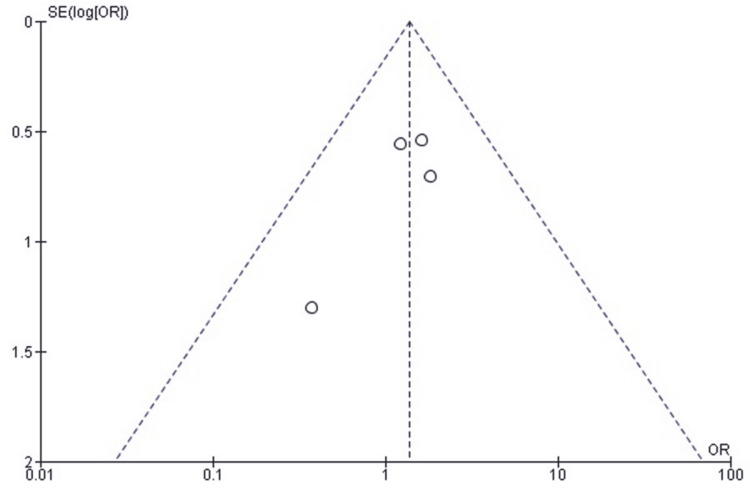
Funnel plot assessing publication bias for studies comparing SF and SCF regarding full ROM recovery ROM, range of motion; SCF, screw fixation; SF, suture fixation

A funnel plot was used to evaluate publication bias, and the distribution of study effect estimates was approximately symmetrical, with minor asymmetry likely due to the differences in standard error and sample size rather than selective reporting or small-study effects (Figure 13); Egger’s regression test showed no statistically significant evidence of publication bias (p > 0.05).

Discussion

A review comparing SF and SCF for pediatric TEFs found that although both fixation techniques resulted in satisfactory union and functional outcomes, SF was associated with fewer reoperations and hardware-related complications [30]. Pooled results showed that SF significantly reduced the rate of reoperation compared with SCF [30,32,33], with reoperation rates of 39% in patients treated with sutures versus 66% in the SCF group [30], 39% versus 49% [32], and 9.4% versus 20.7% for open osteosuture and arthroscopic SCF groups, respectively [33]. A recent meta-analysis by Manaf et al. [26] confirmed these findings, reporting a significantly higher risk of secondary surgery and complications with SCF. However, Ercan et al. [31] did not report any reoperations in either group when using headless compression screws, suggesting that modern screw designs may reduce hardware-related issues.

SF also showed a clear advantage in reducing hardware removal, with 62% of patients in the SCF group requiring implant removal compared with 9% in the SF group (Callanan et al. [30]), 29% versus 11% (Granadillo [32]), and 10% versus 0% (Jääskelä et al. [33]). Ercan et al. [31] found that headless screws minimized this issue, achieving results similar to SF. This reflects inherent design differences between the methods: screws, particularly metallic ones, can protrude into the joint, causing impingement or irritation, whereas sutures are intra-articularly smooth and rarely require removal. Across most studies, SF consistently avoided the need for secondary hardware removal procedures.

There was no significant difference in postoperative knee stability between the fixation techniques. Fourteen percent of patients in both groups reported subjective instability, with equivalent objective stability [32]. Callanan et al. [30] found no difference in laxity or reinjury rates, and Ercan et al. [31] reported symmetric stability with side-to-side KT-1000 differences below 3 mm for both groups. Jääskelä et al. [33] also found no differences in anterior tibial translation or pivot-shift test outcomes. Biomechanical studies by Thome et al. [9] demonstrated similar stiffness under cyclic loading for both techniques, and Ye et al. [22] reported equivalent displacement after mechanical testing.

The rate of arthrofibrosis requiring intervention was not significantly different between the fixation methods. Postoperative stiffness occurred in 24% of suture-treated patients and 31% of screw-treated patients [30], while Granadillo [32] reported arthrofibrosis in 8% of the suture group and 14% of the screw group. Ercan et al. [31] and Jääskelä et al. [33] found no differences between groups. These findings support Coyle et al.’s [14] hypothesis that arthrofibrosis is more closely associated with prolonged immobilization and delayed rehabilitation than with the type of fixation.

RTS rates were generally higher and faster with SF. Jääskelä et al. [33] reported a median return at eight weeks for osteoSF compared with 21 weeks for SCF. Callanan et al. [30] reported 91% versus 74%, and Granadillo [32] reported 100% versus 93% for suture versus SCF, respectively. Ercan et al. [31] found all patients in both groups returned to activity by six months, suggesting parity in long-term outcomes. The earlier return with SF may reflect reduced hardware irritation and earlier initiation of motion exercises. Wien Aryana [11] similarly noted that suture constructs do not impede early rehabilitation.

Excellent recovery of ROM was observed in both fixation methods. Callanan et al. [30] reported 76% full ROM in the suture group versus 66% in the screw group. Ercan et al. [31] found nearly identical outcomes, and Granadillo [32] and Jääskelä et al. [33] reported comparable flexion-extension arcs at final follow-up. These findings align with biomechanical studies by Ye et al. [22] and Thome et al. [9], showing similar fixation stiffness and displacement resistance. Callanan et al. [30] noted minor extension loss in a few screw-treated cases, likely due to notch impingement or fragment elevation.

Limitations

This systematic review has limitations due to heterogeneity in study design, sample size, and follow-up duration among included studies. Most studies were retrospective, which may introduce bias. Variations in surgical techniques and postoperative rehabilitation protocols may also influence outcomes. Nevertheless, the consistency of findings across studies supports their validity.

## Conclusions

Our meta-analysis indicates that SF is superior to SCF regarding reoperation rates, hardware-related procedures, and RTS outcomes, while showing no significant differences in union rates, recurrent instability, or knee ROM. These findings suggest that SF may be the preferred technique for select pediatric patients, particularly when aiming to minimize implant burden and avoid secondary procedures. However, high-quality, prospective, and comparative studies with standardized surgical techniques and outcome measures are limited. Further research is needed to definitively establish the optimal role of SF in managing pediatric TEFs.
